# Design and Modeling of Light Emitting Nano-Pixel Structure (LENS) for High Resolution Display (HRD) in a Visible Range

**DOI:** 10.3390/nano10020214

**Published:** 2020-01-27

**Authors:** Tsion Eisenfeld, Avi Karsenty

**Affiliations:** 1 Advanced Laboratory of Electro-Optics (ALEO), Department of Applied Physics/Electro-Optics Engineering, Lev Academic Center, Jerusalem 9116001, Israel; tsioneisenfeld@gmail.com; 2 Nanotechnology Center for Education and Research, Lev Academic Center, Jerusalem 9116001, Israel

**Keywords:** nano pixel, light emitting diode (LED), enhanced efficiency, augmented reality (AR), virtual reality (VR), modeling

## Abstract

LENS (Light Emitting Nano-pixel Structure), a new nano-metric device, was designed, simulated, and modeled for feasibility analysis, with the challenge of combining high resolution and high brightness for display, essentially adapted for Augmented Reality (AR) and Virtual Reality. The device is made of two parts: The first one is a reflective nano-cone Light Emitting Device (LED) structure to reduce the Total Internal Reflection effects (TIR), and to enable improved light extraction efficiency. The second part is a Compound Parabolic Concentrator (CPC) above the nano-LED to narrow the outgoing light angular distribution so most of the light would be “accepted” by an imaging system. Such a way is drastically limiting any unnecessary light loss. Our simulations show that the total light intensity gain generated by each part of the pixel is at least 3800% when compared to a typical flat LED. It means that, for the same electrical power consumption, the battery life duration is increased by 38. Furthermore, this improvement significantly decreases the display thermal radiation by at least 300%. Since pixel resolution is critical to offer advanced applications, an extensive feasibility study was performed, using the LightTools software package for ray tracing optimization. In addition to the simulation results, an analytical model was developed. This new device holds the potential to change the efficiency for military, professional and consumer applications, and can serve as a game changer.

## 1. Introduction

### 1.1. The Need for a Nano-Display

In the age of Full High Definition screens, the largest companies are engaged in hard technological battles in order to achieve ever larger image resolutions [1]. The sharper the image, the more it appears to be real, thus reaching the limits of the eye’s visual acuity, less than 1 arc minute per line pair [2]. As part of the candidate applications, one can find the living room’s and computer’s screens, but also smartphone’s screens and, more recently, headsets of virtual reality (oculus) and augmented reality (Lumus [3], Microsoft HoloLens [4]).

There is a clear need for Nano-Display. If it is true that for the moment that the pixels are the size of about 50 to less than 10 microns [5] depending on the application, the day will come when the thirst for resolution will lead us to develop pixels smaller than a micrometer. Researchers will continue to push manufacturing limits even further. This is called nanotechnology. In fact, nanotechnology has been able to meet the highest requirements of transistor manufacturers (Intel, AMD, Qualcomm, ARM, Nvidia...) by allowing component manufacturing of a few nanometers in width [6]. Nanotechnology has allowed to considerably increase the number of these small precious devices, and thus to constantly improve the computing power of our computers [7]. Today, Extreme UV photolithography manufacturing method has dramatically decreased the size of the transistors. Thus, applying to the world of display, nanotech has the potential to multiply the number of pixels on the same surface size as for transistors on a chip. However, for what reason would we want to start this nano-pixel race? Is the resolution of displays not already sufficient? Is the quest at the highest resolution not vain and useless? What characterizes high technology is the ability to offer new products and open up new horizons that until now had no particular need. Who would have thought that we would need a mobile phone that can photograph, film, and visualize everything directly on a high-quality color screen and even share it with family without taking a step?

The technological advance indisputably sets the pace for manufacturers and consumers by offering them the products that, in addition to entertaining, advance the well-being of the human race and create new tools that are constantly improving. Today, it is acceptable to forecast that the future applications, based on very high-resolution screens, will certainly be optical systems such as Augmented Reality (AR) glasses and Virtual Reality (VR) headsets, where a high pixel resolution is required in very small size displays. For example, nowadays, the Himax company manufactures micro-display specially designed for headsets and sharing a resolution of 1024 × 768 pixels in a display size of 0.45’’ [8]. The AR/VR market is estimated to exceed $100 billion in the coming years [9]. Such AR glasses directly project into the eye large and versatile quantities of data, without having to look at a smartphone screen. In addition, 3D objects will sneak into our field of vision and will be an integral part of our environment [10]. However, in order to reach such a milestone, AR glasses developers will have to find a way to make their eyewear aesthetic, lightweight, and easy to wear. In addition, the image quality will be paramount to ensure the best possible experience for the user [11].

Several parameters are essential to maintain a reasonable Head Mounted Displays’ (HMD) form factor while maintaining a good image quality. The screen or micro-display must be of the smallest possible size while ensuring sufficient resolution. Today, consumers’ resolution requirements are Full High Definition (FHD) and more. How to fit such a quantity of pixels on a surface of a few millimeters? Obviously, nanotechnology could then give a solution as it does for electronic chips. Pixels, smaller than one micrometer, will keep the same number of pixels as for a high-resolution computer screen but on a much smaller surface. As a result, the optical system responsible for projecting the image into the user eye would see its size reduced, substantially making these glasses less cumbersome.

Nano displays are a game changer which enables reaching higher image resolution while maintaining very compact display panel dimensions. This is critical to ensure a good image quality in an acceptable form factor. Generally, the more the pixel count grows, the more the screen size expands. To shrink display dimensions, pixels need to reduce its size. Sub-micron pixels, in the nano scale, are without a doubt the holy grail of compact displays. Some future technologies will be completely dependent on nano pixels such as augmented reality contact lenses. Nano displays will ensure a small enough form factor, very high pixel resolution aside, with minimum thermal radiation.

### 1.2. Augmented and Virtual Reality (AR/VR)

Head Mounted Displays headsets and Augmented Reality glasses and even smart contact lenses will sooner or later become a new High-Tech revolution, as it happened with smartphones two decades ago. Such a new reality will change the way we communicate, drive, and look for useful information. The consumer will find himself in a hybrid world where three-dimensional objects and persons will be part of its real environment and background. The main challenge is that the created and projected artificial 3D objects and persons will be indistinguishable from real ones. Otherwise, the consumer immersion sensation will be compromised. Optical scientists and physicists are working hard to realize aesthetic Augmented Reality glasses and headsets with acceptable image quality. Today, AR optical engines have lower contrast ratio, colors purity, brightness, and less resolution than common TV and computer screens. Furthermore, their large dimensions make them look ugly and displeasing to the common consumer, severely limiting their market applications. Moreover, a high illumination power requirement makes batteries endurance ephemeral, a couple of hours in the best case, and makes them look big. AR glasses or headsets will need to look the same or thinner than large sunglasses in order to be acceptable for the consumer market. In this research, we intend to address these requirements by developing a nano-display technology, gathering all the advantages and enabling a new kind of consumer experience. In other words, the challenging aim of this research is to develop a new designed display, sharing smaller dimensions while maintaining similar or better resolution, higher pixel extinction capability than Liquid Cristal based micro-displays, augmented light efficiency, and higher brightness than OLED based micro-displays. A first publication of such Super-High Intensity Nano-Emitting (SHINE) pixel only was recently presented [12].

### 1.3. LENS Proposed Solution

Our innovation seeks to develop a new kind of display, essentially adapted for Augmented and Virtual Reality (AVR) application, where the pixel’s resolution is critical, in order to offer a valuable user-experience for military, industry and consumer applications. To be suitable, such a display requires a high resolution while maintaining small dimensions. The proposed monochromatic green Nano-LED is based on InGaN/GaN materials. Indeed, direct GaN-based green LED emits a narrower spectrum than the phosphor converted green LED. As a result, the direct GaN green has a higher purity green light [13]. Furthermore, the display brightness [14] and its power efficiency (Lumens/Watts) are two essential parameters allowing, or not, outdoor applications and long battery life. Since the thermal dissipation remains a complicate exercise in very compact systems, increasing the light efficiency enables reducing the display working temperature [15]. 

Our proposed solution to the power efficiency issue is twofold: First, a Reflective Nano Cone LED structure to reduce the Total Internal Reflection effects (TIR), and to enable improved light extraction efficiency—secondly, a Compound Parabolic Concentrator (CPC) above the Nano-LED to narrow the outgoing light angular distribution so most of the light would be “accepted” by an imaging system. Such a way is drastically limiting any unnecessary light loss. It was demonstrated in our simulations that, when combined, the cone Nano-LED and the CPC improve the power efficiency by around 3800% when compared to a conventional flat shape LED. The design of such a Reflective Cone Nano-LED is presented. As part of this research, the LightTools software package was used for the ray tracing optimization, and in addition to the numerical simulation results, an analytical model was developed, as presented below.

## 2. Device Concept and Structure 

### 2.1. Existing Technologies in the Industry

Before presenting our proposed solution, it is important to review the main four micro-display existing industrial technologies, as well as the recent progresses at the academy [16]:

1. The Liquid Crystal Display (LCD) [17] is a flat-panel display or other electronically modulated optical device which uses the light-modulating properties of liquid crystals. Liquid crystals do not emit light directly, instead using a backlight or reflector to produce images in color or monochrome.
2. The Liquid Crystal-On-Silicon (LCOS) [18] technology has been developed for many years for image and video display applications. This technology combines the unique light-modulating properties of Liquid Crystal (LC) materials and the advantages of high-performance silicon Complementary Metal Oxide Semiconductor (CMOS) technology through dedicated LCOS assembly processes.
3. The Organic Light-Emitting Diode (OLED) [19]: In organic light-emitting diodes, the electro luminescent material comprising the emissive layer of the diode is an organic compound. The organic material is electrically conductive due to the delocalization of electrons caused by conjugation over all or part of the molecule, and the material therefore functions as an organic semiconductor. Through electron–hole recombination, a high-energy molecular state is formed. This state called exciton, behaves like a single molecule with high energy, and generates light after an exciton lifetime period.
4. The Digital Light Processing (DLP) [20]: The Digital Mirror Device (DMD) is a Micro Electro Mechanical System (MEMS) device invented in 1987. The DMD is designed for projection usage, where the tilting mirror pixels reflect the light out of the projection lens. Thus, the DMD generates a large, bright and high contrast image in comparison to other display technologies as LCOS, LCD, or OLED displays.

### 2.2. Recent Progress in Academy

These last few years, several research teams have developed different nano-pixels technologies: A team led by Oxford University scientists explored the link between the electrical and optical properties of phase change materials (materials that can change from an amorphous to a crystalline state). They found that, by sandwiching a seven-nanometer thick layer of a phase change material between two layers of a transparent electrode, they could use a tiny current to ‘draw’ images within the sandwich stack [21]. At McGill University and McMaster University, multicolor single InGaN/GaN dot-in-nanowire Light Emitting Diodes (LEDs) were fabricated on the same substrate using selective area Epitaxy. It is observed that the structural and optical properties of InGaN/GaN quantum dots depend critically on nanowire diameters [22]. A research team from Illinois University developed a hierarchical multicolor nano-pixel matrix formed by coordinating luminescent metal ions to a conjugated poly (4′-octyl-2′,6′-bispyrazoyl pyridine) film via contact printing [23]. At the National Chiao Tung University in Taiwan, a Nano-Ring Light-Emitting Diodes (NRLEDs) with different wall width (120 nm, 80 nm and 40 nm) were fabricated by specialized nano-sphere lithography technology [24]. 

### 2.3. LENS Proposed Solution: Architecture, Design and Added Values

**Figure 1:**
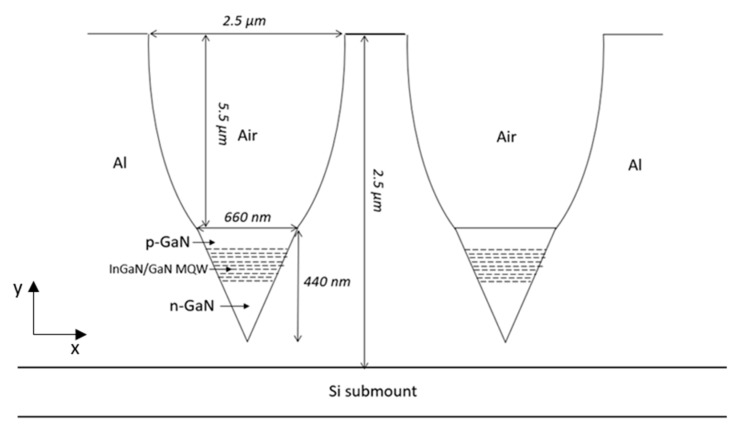
Presentation of a cross section of an array of two adjacent LENS devices. The design includes a Reflective Coned Nano-Green LED with a Compound Parabolic Concentrator (CPC). The figure is voluntarily not to scale in order to present the new concept.

The LENS proposed solution is made of an array of Green Nano-LEDs with sub-micron dimensions, 660 nm diameter. LENS itself is made of two parts: A reflective conical nano-pixel structure and a light condenser. This reflective conical structure permits narrowing the outgoing light angular distribution and thus limits total internal reflections on the output surface. As a result, more light is allowed to exit the pixel as shown in Figure 1. 

**Figure 2:**
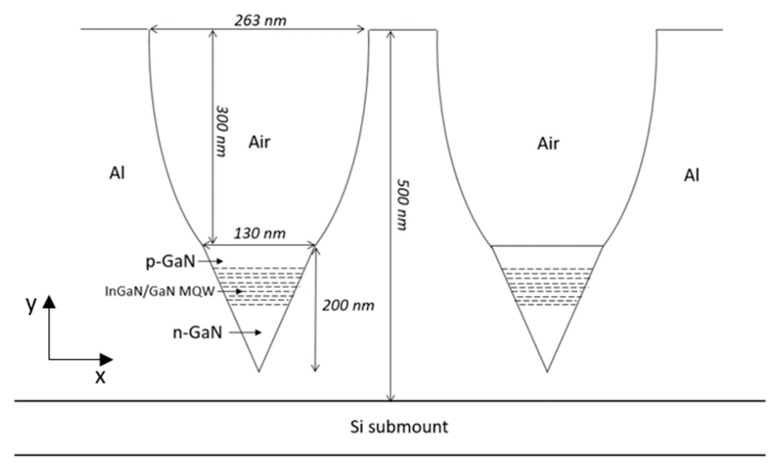
Presentation of a cross section of an array of two adjacent LENS devices with reduced dimensions. The design includes a reflective Coned Nano-Green LED with a Compound Parabolic Concentrator (CPC).

As part of future research, sub-wavelength dimensions green LED will be investigated, with a 130 nm diameter. Again, a reflective conical nano-pixel structure to allow a better Light Extraction efficiency and a light condenser will be considered as shown in Figure 2.

There are several bottlenecks that the present study aims to solve or contribute for a better solution: Shrinking the LED dimensions avoids internal light absorption, essentially from the quantum well. In our study, a proposed 5 nm thickness quantum well absorption is as small as 0.5%. Furthermore, designing a conical shape LED to recycle TIR trapped rays, greatly contributes in multiplying the Light Extraction Efficiency in comparison to a flat shape LED. Lambertian light angular distribution in a regular LED results in unnecessary light loss. Indeed, most of the light shining from a flat LED won’t be accepted by an optical system with smaller numerical aperture. Thus, adding a Compound Parabolic Concentrator on the LED’s top allows controlling accurately the output Light beam angular distribution. This way, light is injected into an optical system with little or no light loss.

## 3. Methods

### 3.1. Monte Carlo Ray Tracing Using LightTools Simulation Software

An optical condenser element has been designed in order to reduce the angular distribution of the Nano-LED pixel. In this case, the Monte Carlo ray tracing method is used to understand and determine a way to collect rays going out from the Nano-LED and to shape the outgoing beam with a reduced numerical aperture. An optimization method is used to design the best condenser. 

LightTools optical software has been selected to generate our Monte Carlo simulations. Built-in tools and modules have been used to optimize and analyze our investigated pixel. LightTools is a software especially adapted for non-sequential ray tracing, analysis, and optimization. Variables, constraints, and merit functions are set while proprietary algorithms within the LightTools optimization engine will minimize the merit function by changing the defined variables and simultaneously satisfying the specified constraints. Different kinds of condensers were considered, for example conventional spherical lens, a-spherical lens, freeform shape, reflective walls, and total internal reflection lens. Furthermore, Boolean operations can be done to make custom elements. This software allows us to define any material with specific optical parameters from scientific literature: 1. Absorption Spectrum, 2. Emission Spectra, 3. Intensity Distribution and other simulation parameters such as the number of rays propagating from Nano-LED regions. Then, we will be able to realize a realistic model of the LED and its material properties—in LightTools, when the distribution from a light source can be simulated by tracing Monte Carlo rays. These rays are accumulated on receivers, and are used to compute illumination analysis metrics such as: Illuminance (spatial distribution of power), Intensity (Angular distribution of power), and Luminance (both angular and spatial components). When rays are collected on a receiver, the data are usually divided into rectangular grids to analyze ray data. Data can be displayed as numbers/color maps.

### 3.2. Physical Simualtions vs. Geometry Ray Tracing

Monte Carlo ray tracing is a geometric approximation commonly used in optics. This method of light propagation ignores physical effect as wave effects, electrical behavior of the implicated materials, and more. When reducing the investigated element dimensions below the light wavelength scale, such approximations are useless. In the nano-scale, Maxwell equations are the ones able to simulate the light behavior. Furthermore, diode junction analysis cannot be simulated with a ray tracing software. As part of all the physical aspects of a light-emitting diode, we find the spectral spectrum of an active layer, the current–voltage curve, and the energy diagram. Thus, the necessity of complementing physical software, which are capable of running physical equations and algorithms, will become necessary in the future. 

## 4. LightTools—Ray Tracing Results

### 4.1. Reflective Nano-Cone LED

#### 4.1.1. Structure, Dimensions, and Light Path

**Figure 3:**
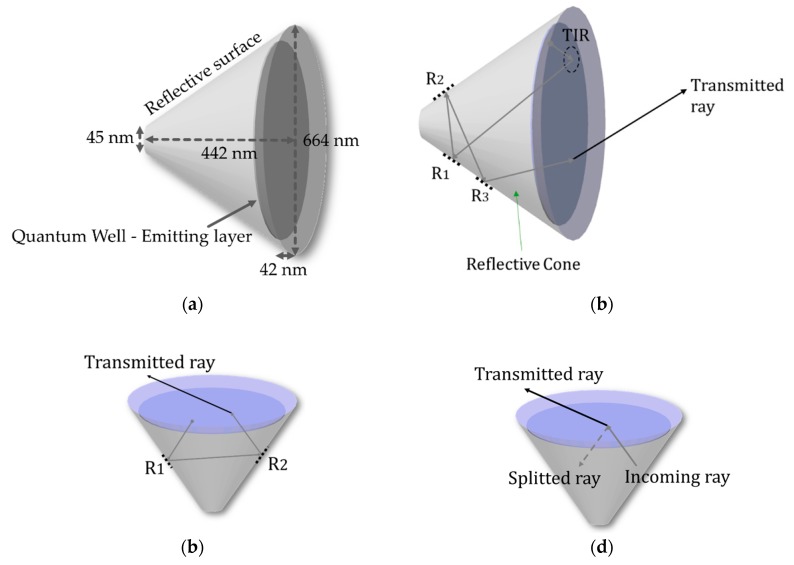
(**a**) LENS pixel Cone LED structure; (**b**) a typical ray path inside a reflective conical LED obtained with LightTools software; (**c**) inwards emission light path; (**d**) outgoing ray split by Fresnel reflection.

The first floor of the investigated pixel is a light-emitting diode which emits a green light by electron/hole recombination. The p-type/n-type (PN) junction is made of lnGaN/GaN material with a 525 nm wavelength spectral peak. The GaN refractive index equals 2.4, which is much higher than an amorphic optical material, typically with a refractive index equal to 1.5168 as for BK7 glass material [25]. The structure of the conical LED is presented in Figure 3a. The emitting junction is a circular area located at a distance of 42 nm from the outgoing surface as shown in the same figure. The light emitting area is installed in a conical cavity with reflective outer surfaces allowing rays to be reflected out of the LED. The dimensions chosen for our simulations are presented in Figure 3a where the conical base diameter is 664 nm, and the overall height 442 nm. This 3/2 structural ratio is the result of our optimization effort. The Merit function was built such that the maximum light power could exit the conical structure, which is to say in order to get the best light efficiency possible. The structure’s top is truncated in order to meet realistic manufacturing constraints since a perfect top is not realistic. Such a relatively small truncation has no significant performance repercussion. As Snell’s law describes it, when rays try to pass from an incident medium to another with a lower Refractive Index (RI), only a few portions of light will pass and the rest will be reflected back by what is call Total Internal Reflection (TIR). Rays with angles smaller than the material critical angle will manage to escape. Instead, rays having higher angles than the critical ones will experience a total internal reflection. We understand that the main challenge for a Light Emitting Diode (LED) is the light efficiency. Since the material used, lnGaN/GaN, has a high refractive index, only a relatively small angular range will manage to escape outside the LED as shown in Figure 3b–d.

#### 4.1.2. Light Extraction Efficiency (LEE)

The Light Extraction Efficiency (LEE) is a crucial parameter. In order to enhance it, there is a need for shrinking the LED dimensions in order to avoid internal light absorption and optimize the conical shape to recycle rays. This greatly contributes to multiplying the Light Extraction Efficiency. In such geometry, the light is forced to exit the LED. If it does not succeed at the first tentative, the light will be reflected back and forth again and again until it finally exits the structure. The only parameter that can affect the efficiency is the reflective wall and internal materials absorption. This is where nano scale comes to shrink the distances that light travels, avoiding any significant absorption.

**Figure 4:**
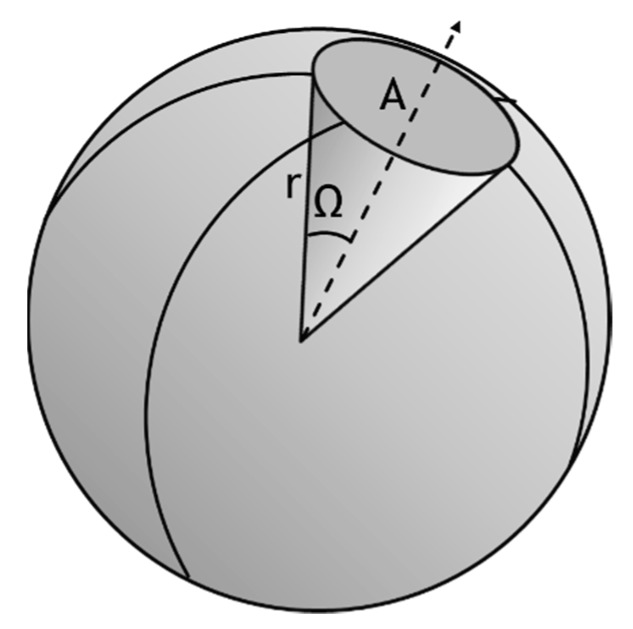
Projection of a cone onto a sphere.

The total internal reflection constraint makes a GaN based LED light extraction very challenging. Indeed, GaN’s RI is much higher than air’s Refractive index reducing significantly the critical angle value. The refractive indexes used in this research for the three layers, are respectively 2.54 (Quantum Well), 2.45 (P-GaN), and 2.42 (N-GaN). The corresponding absorption coefficients are 1 µm^−1^ for the Quantum Well, and 0 for the p-type Gallium-Nitride (P-GaN) and n-type Gallium-Nitride (N-GaN) layers. Then, the theoretical light extraction efficiency of a flat LED can be given by the ratio of the solid angles of a cone with an apex of Ω = 2 × θ_c_ and a sphere solid angle of 4π, as shown in Figure 4:

For a Lambertian angular distribution emitter:

(1) $$\text{LEE lambertian emission}=\frac{2\pi \left(1-\cos^{2}\theta_{c}\right)}{4\pi }\times 100$$

where Ө_c_ = 24.6° for a GaN material. Thus, the resulting theoretical light extraction efficiency is 8.66%. For a uniform angular distribution emitter:

(2) $$\text{LEE uniform emission}=\frac{2\pi \left(1-\cos\theta_{c}\right)}{4\pi }\times 100$$

The resulting theoretical light extraction efficiency is 4.54%.

Furthermore, in order to elaborate an accurate theoretical LEE model, Fresnel reflections should be implemented to our equation. Indeed, rays coming out from the LED are divided in two segments. One segment exits the LED and the other one is reflected back. The energy of the reflected segment and the transmitted one are a function of the Angle Of Incidence (AOI) and of the Refractive index (RI) of both of the medium n_1_ and n_2_. The following are Fresnel reflections equations [26] as shown below:

(3) $$r_{s}=\frac{n_{1}\cos\theta_{i}-n_{2}\cos\theta_{t}}{n_{1}\cos\theta_{i}+n_{2}\cos\theta_{t}}$$

(4) $$t_{s}=\frac{2n_{1}\cos\theta_{i}}{n_{1}\cos\theta_{i}+n_{2}\cos\theta_{t}}$$

(5) $$r_{p}=\frac{n_{2}\cos\theta_{i}-n_{1}\cos\theta_{t}}{n_{2}\cos\theta_{i}+n_{1}\cos\theta_{t}}$$

(6) $$t_{p}=\frac{2n_{1}\cos\theta_{i}}{n_{2}\cos\theta_{i}+n_{1}\cos\theta_{t}}$$

where: r_s_ is the light S polarization reflection, r_p_ for P polarization, t_s_ is the S polarization transmission, and t_p_ the P polarization one. The unpolarized transmission for a specific AOI is then given by:

(7) $$t_{\mathrm{unpolarized}}=t_{s}+t_{p}$$

Then, the unpolarized transmission for a range of AOI is given by:

(8) $$\int_{0}^{\theta c}t_{\mathrm{unpolarized}}$$

The updated LEE formula is for a Lambertian angular distribution emitter:

(9) $${\text{LEE lambertian}}^{\prime }=\int_{0}^{\theta c}t_{\mathrm{unpolarized}}$$

**Figure 5:**
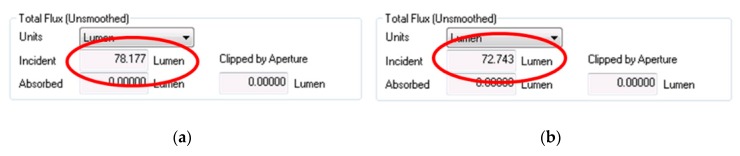
LightTools Light Extraction Efficiency results. (**a**) results for Silver; (**b**) results for Aluminum.

The expected Light Extraction efficiency with Fresnel reflection took into account is then LEE Lambertian = 6.9%. Simulation with LightTools software have shown very similar results with LEE Lambertian = 8.6 % and LEE Lambertian = 6.85%. Side walls material absorption is also a critical parameter influencing the real possible Light extraction efficiency. Two materials have been investigated, Silver and Aluminum. Silver has the highest possible reflection in the visible range, but it is more expensive. However, Aluminum is a much cheaper material sharing a higher light absorption As a result, it can be seen that LightTools simulation LEE results (Figure 5) are better for the Silver material than for Aluminum. For Silver material, the cone has ~78% efficiency (Figure 5a), and for Aluminum material ~73% (Figure 5b). This is an improvement of 900% in comparison to a flat LED.

#### 4.1.3. Emitter Position vs. LEE

**Figure 6:**
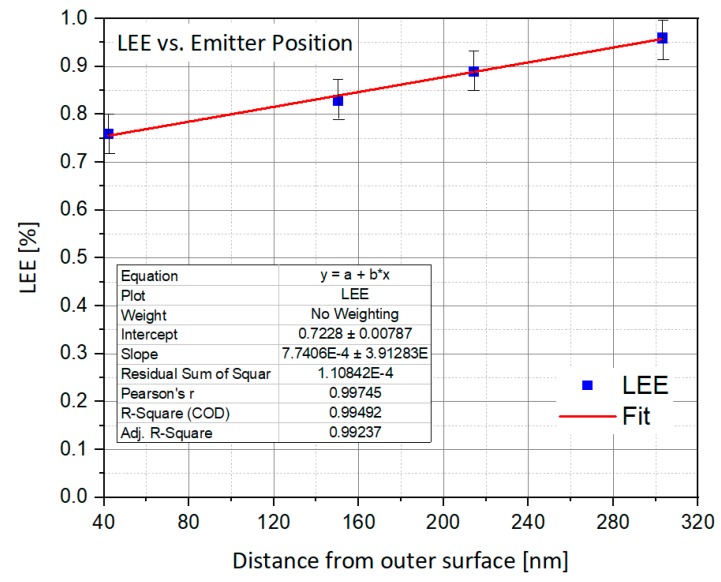
Light Extraction Efficiency vs. Emitter Position.

**Figure 7:**
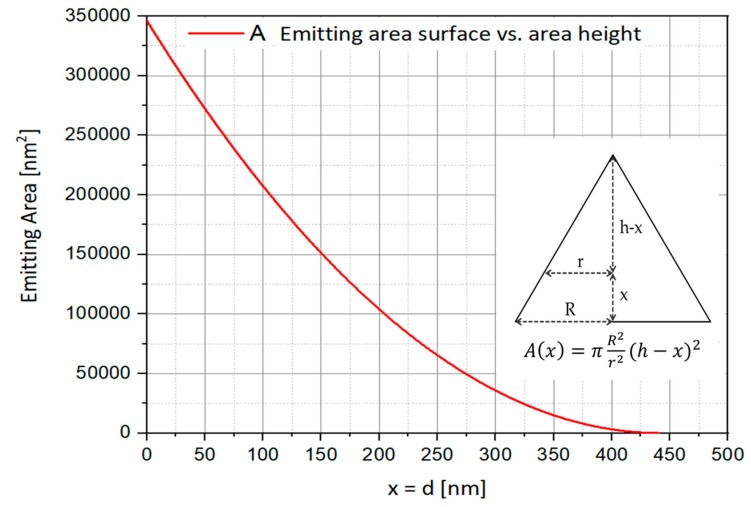
Emitting area surface vs. area height.

As the layer is pushed away from the exit, the LEE improves. This is explained by the fact that, in these conditions, the number of reflections on the side walls is decreasing as a function of the layer location as shown in Figure 6. In the other hand, pushing back the emitting surface shrinks it because of the cone structure. As we reach the top of the cone, the diameter section decreases. This way, the total brightness is much reduced. Then, a distance of 42 nm seems to be an acceptable solution, since the emitting area decreases much faster than the efficiency increases, as shown in Figure 7.

#### 4.1.4. Outgoing Light Angular and Spatial Distribution

**Figure 8:**
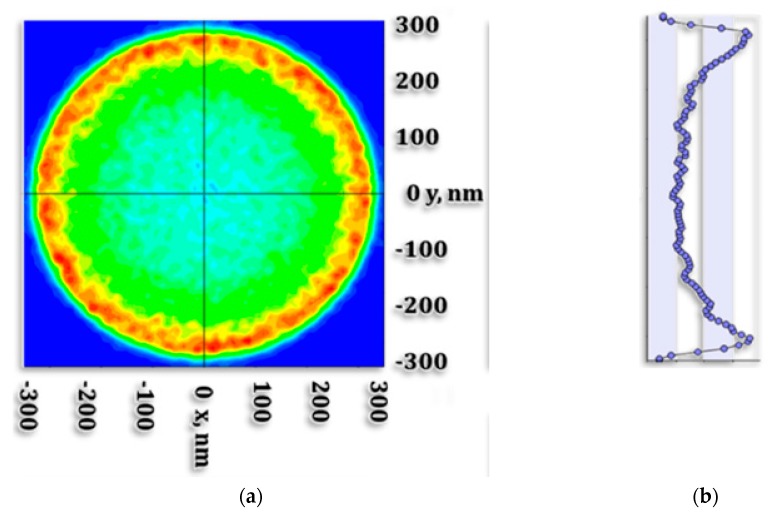

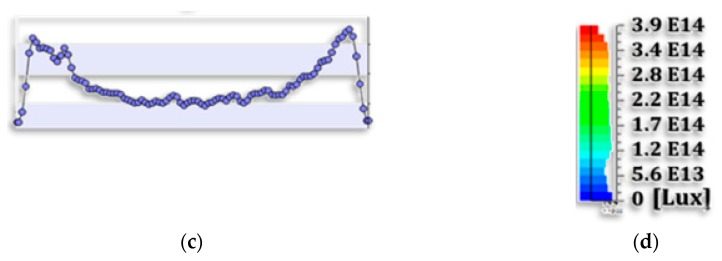
Outgoing Cone light spatial distribution obtained with LightTools software. (**a**) cone light; (**b**) *y*-axis distribution; (**c**) *x*-axis distribution; (**d**) colors Legend in Lux units.

In order to analyze the light distribution at the cone exit aperture, an intensity receiver and an Illuminance receiver were placed after the exit, in ambient air. Each receiver displays its own results. The Illuminance information displays the light power as a function of the ray incidence location on the receiver. Power units are in Lux. The intensity receiver displays the power of the rays incoming on the receiver surface as a function of their angle of incidence. The resulting Illuminance spatial distribution is presented in Figure 8. Units are in Candela. The cross section shows a batwing like distribution where the power is more concentrated at the edge of the aperture. A layout analysis allows us to visualize the concerned rays contributing to the central area and to the aperture edges. In the central area, rays are emitted outward, then reflected back by TIR and reflected outwards again. Some rays are reflected by the top of the cone, which is a plane surface.

#### 4.1.5. High Brightness Conical Emitter Layer

**Figure 9:**
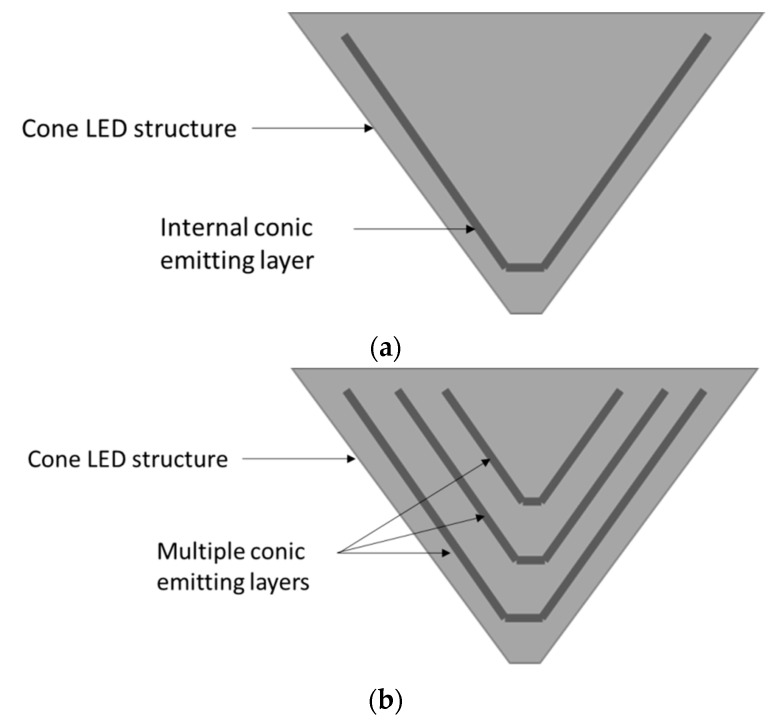
LED cone. (**a**) LED cone with internal conical emitting layer; (**b**) LED cone with multiple sub-cone emitting layers.

**Figure 10:**
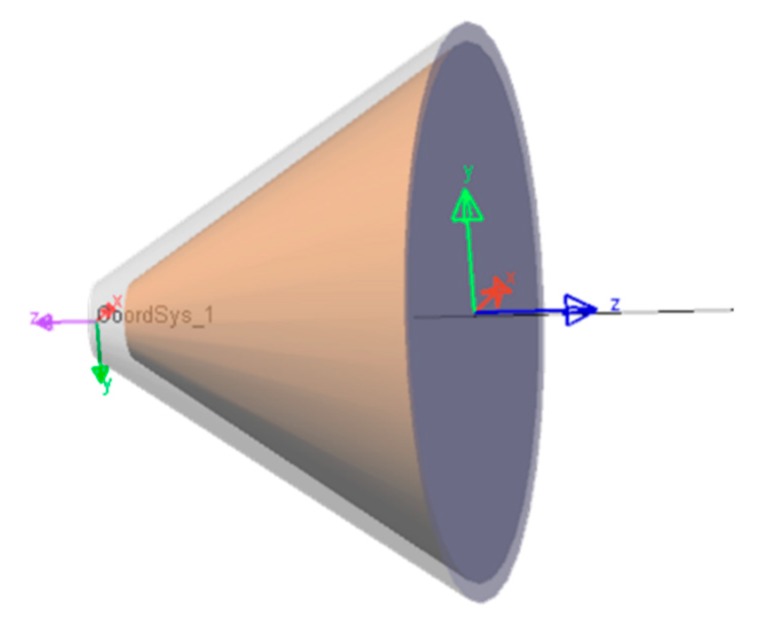
LightTools 3D Model of an LED cone with internal conical emitting layer.

The usual brightness in daylight is around 3000 nits [14]. In an HMD, to be visible in daylight conditions, the image should be projected with a similar brightness. To ensure the highest brightness possible, the emitting layer area should be as large as possible in order to increase the amount of light emitted. The increased emitting area improves the maximum brightness by the area enlargement factor. The idea is to follow the conical structure with the emitting layer in order to spread it over the largest area possible (Figure 9a). This process can be done several times to increase the emitting area even more (Figure 9b). The LEE is not significantly affected by this increased emitting area. Still, our LightTools simulations (Figure 10) show a result of 79% LEE, very similar to the circular emitting layer configuration seen before. The total gain in emitting surface is the ration of the circular surfaces area described earlier and of the conical one. Considering that the cone dimensions are as described earlier, the resulting emitting surface gain is:

(10) $$\mathrm{Gain}=\frac{{\pi r}^{2}}{\pi r\;\left(r+\sqrt{h^{2}+r^{2}}\right)}=\frac{41.59\times 10^{-2}}{13.05\times 10^{-2}}=3.19$$

Then, the brightness also should be improved by ~300% accordingly to the updated LEE. The gain could be improved even more with additional conical emitting sub-layers.

### 4.2. Compound Parabolic Concentrator (CPC)

The Compound Parabolic Concentrator is the complementary part of the pixel. The first part is the light emitting diode with a conical structure permitting to extract light more efficiently, as described earlier. The second part function is to narrow the angular distribution of the light beam. This is required to ensure that the light emitted from the pixel fits the numerical aperture of a desired optical system. An optical system has an acceptance angle into which light can propagate through the optics. Any ray having an angle of propagation higher than the optical system angle of acceptance, will not succeed in propagating inside the system and will be a loss. If the outgoing Cone LED rays could be shaped to have a narrower angular distribution, less rays will be lost reducing—then the waste of power, the overheating of the display, and, as a result, the battery power consumption and its overheating as well. Inside the CPC, light is reflected by a parabolic shaped wall by total internal reflection and eventually exits the structure.

**Figure 11:**
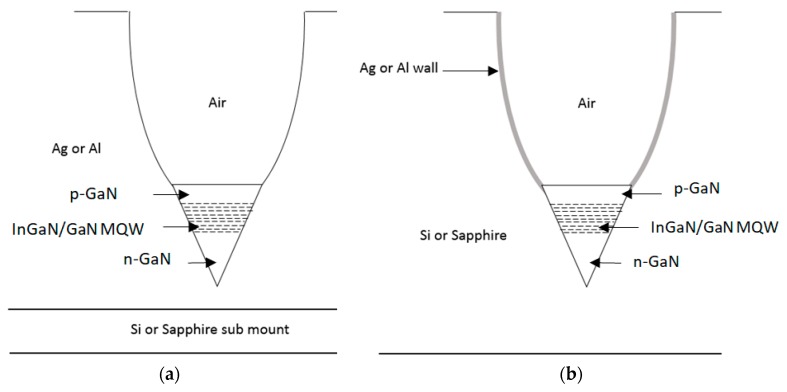
Views of the CPC structure. (**a**) a reflective cavity into Ag or Al material; (**b**) a CPC with Ag or Al reflective wall inside a Si or Sapphire material.

There are no secondary reflections on the CPC wall, each ray is reflected only once and then exits. If necessary, walls can be made from a reflective material such as Al and Nickel so rays would not be reflected by TIR. This would allow for designing a CPC cavity into any relevant wafer material like Sapphire or Silicon (Figure 11a,b). This possibility is more suitable for manufacturing but is less efficient since the reflecting performances would be affected by the material absorbance when TIR achieves a 100% reflection. Silver or Aluminum absorption is below 10%, making a coated CPC a relevant configuration.

The CPC dimensions are set according to an input angular distribution, an output dimension, and the desire output angular distribution. In our investigation, the CPC has an acceptance angle of ±90° since it collects the light coming out from the Cone LED. The outgoing desired angular distribution, without considering physical diffraction effect, is about ±30°. Then, the dimension and the parabolic profile are given by the set of equations below [27]. The equation of parabola is given by:

(11) $$Y=\frac{x^{2}}{2b\left(1+{\sin\theta }_{c}\right)}$$

The full height of CPC is given by:

(12) $$H=\frac{w}{2}\left(1+\frac{1}{{\sin\theta }_{c}}\right){\cos\theta }_{c}$$

The point on parabola C can be expressed in terms of coordinates, where:

(13) $$X={\mathrm{bcos}\theta }_{c}$$

(14) $$Y=b\left(1-{\sin\theta }_{c}\right)/2$$

Height to aperture ratio is given by:

(15) $$\frac{H}{w}=\frac{1}{2}\left[1+\frac{1}{{\sin\theta }_{c}}\right]\;{\cos\theta }_{c}$$

**Figure 12:**
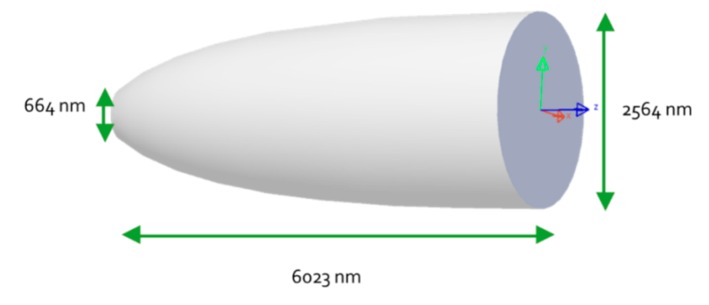
CPC dimensions obtained with LightTools software.

As a result, the dimensions are presented in next in Figure 12.

**Figure 13:**
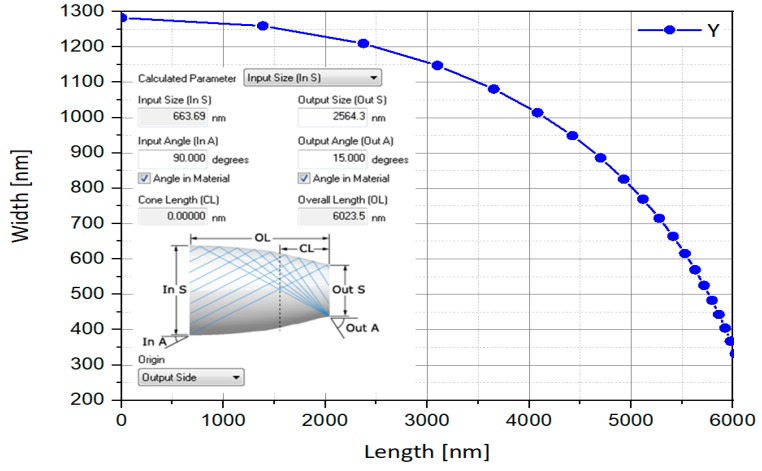
The optimized CPC profile.

Z is the length and Y is the height of a discrete portion of the parabolic shape corresponding to the CPC width. The resulting CPC profile is as in Figure 13.

### 4.3. CPC and Conical LED Assembly

**Figure 14:**
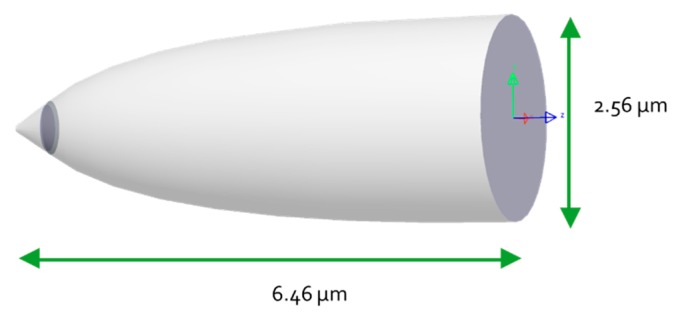
Assembly dimensions obtained with LightTools software.

After being separately optimized, the two parts of the pixel are joined together to achieve the pixel assembly. This assembly is composed by the conical LED with high light extraction efficiency and on its top by the Compound Parabolic Concentrator, whose function is to narrow the beam to best fit the numerical aperture of an optical system. The assembly dimensions are presented in Figure 14. The conical LED outgoing surface area fits the entrance area of the CPC to avoid any loss of light. This way it allows manufacturing the pixel structure as one single cavity instead of manufacturing each part of the pixel cavity in at least two steps. The pixel depth almost reaches a micron and could be even deeper since this dimension doesn’t affect the visible pixel top size, which could remain unchanged. The pixel depth is a degree of freedom, permitting to adapt and optimize the pixel shape, depending on the desired optical system numerical aperture.

#### 4.3.1. Pixel Assembly Ray Path

**Figure 15:**
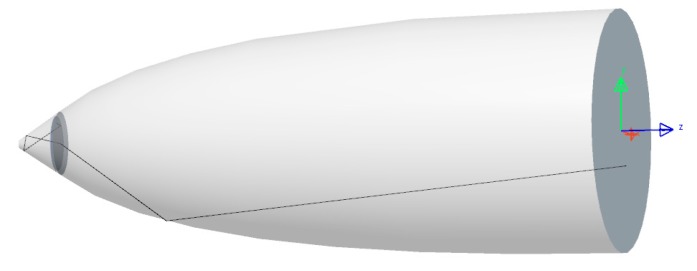
Typical LENS pixel ray path obtained with LightTools software.

The pixel assembly is composed by the Light source, the Cone LED, and the concentrator, the CPC. As predicted, when combined, light is first extracted from the bottom LED and then propagates to the CPC to be concentrated before finally leaving the assembly. The following is a propagating typical ray path (Figure 15), as described earlier.

#### 4.3.2. Outgoing Light Angular and Spatial Distribution

**Figure 16:**
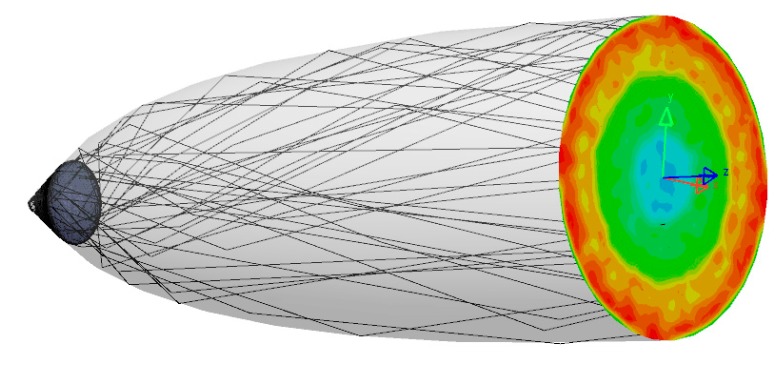
LENS Pixel ray trace with Illuminance pattern output obtained with LightTools software.

**Figure 17:**
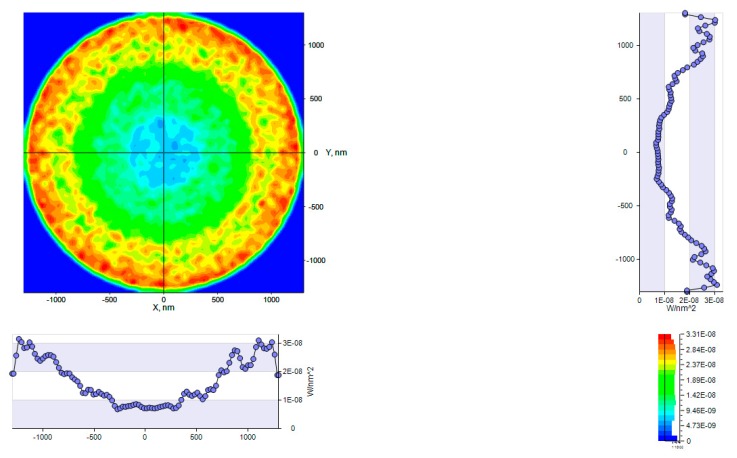
LENA pixel Illuminance spatial distribution output obtained with LightTools software.

The associated Lambertian Cone LED source with the adapted top CPC generates an output spatial distribution as described in Figure 16 and Figure 17.

**Figure 18:**
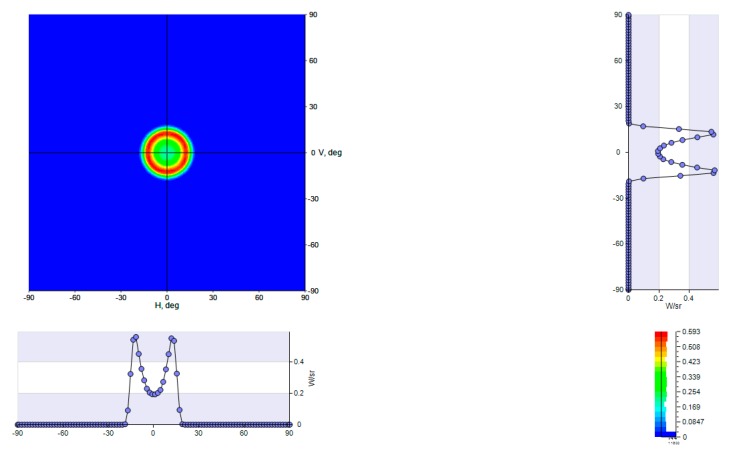
LENS pixel Intensity angular distribution output obtained with LightTools software.

**Figure 19:**
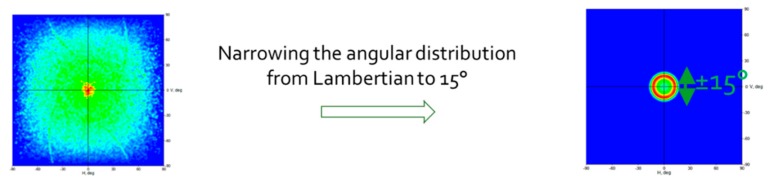
LENS Pixel angular distribution CPC narrowing, LightTools simulation.

As theoretically predicted, the associated Lambertian Cone LED source with the adapted top CPC generates an output angular distribution equal to ±15° as described in Figure 18 and Figure 19.

### 4.4. Complete LENS Pixel Assembly Efficiency Improvement

In comparison to a simple Flat LED, the LENA pixel is several times more efficient. The output efficiency is the result of the Cone LED LEE improvement conjugated to the narrowing effect of the CPC on the output light beam.

#### 4.4.1. Cone LED LEE Improvement

To evaluate the Cone LED light extraction efficiency improvement in comparison to a flat LED, an Aluminum material Cone configuration is chosen. On one hand, it is not the most efficient material, the best being Silver, but, on the other hand, Aluminum is more likely to suit a mass production and industrial manufacturing as its cost is relatively low compared to Silver. Then, the Cone LED Light extraction efficiency improvement relatively to a flat LED LEE is given by:

(16) $$LEE\;Improvement=\frac{Aluminum\;Cone\;LED\;LEE}{Flat\;LED\;LEE}$$

Replacing the obtained values, we get:

(17) $$LEE\;Improvement=\frac{72\%}{6.9\%}=10.43$$

The improvement is ~10 times in comparison to a typical flat LED LEE!

#### 4.4.2. CPC Brightness Improvement

**Figure 20:**
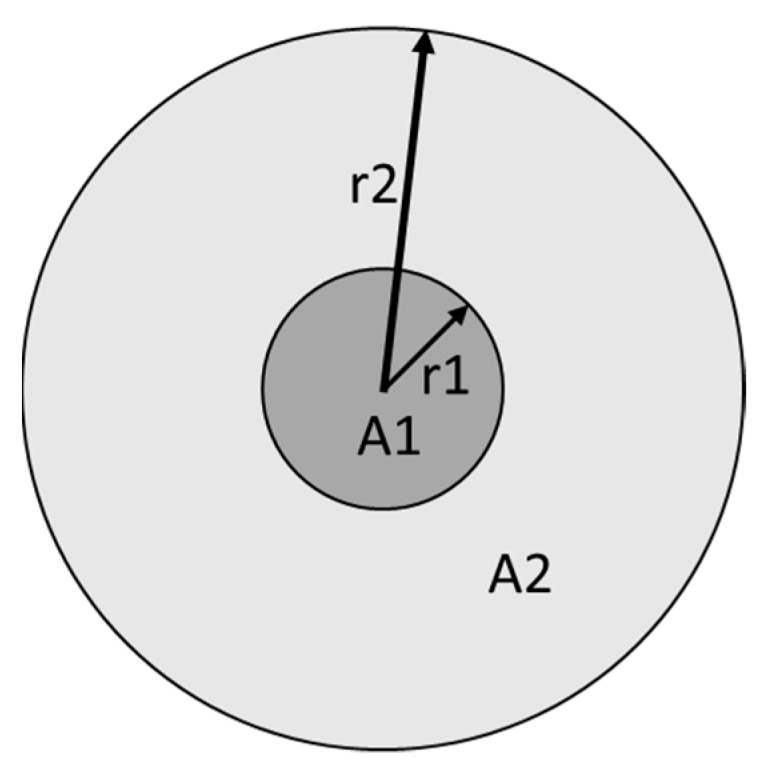
The angular distribution radius of the Cone LED beam r_2_ and the CPC output beam radius r_1_.

Improving the light brightness in an eventual optical system is now possible since the CPC adapts the Nano-LED numerical aperture to the optics acceptance angle. To evaluate the gain obtained by the CPC numerical aperture adaptation, we divide the initial Cone LED angular distribution surface area by the final output angular distribution surface area, and we get:

(18) $$\frac{A_{2}}{A_{1}}$$

where A_1_ is the output angular distribution area and A_2_ the Cone LED as shown in Figure 20.

(19) $$A_{1}=\pi r_{1}^{2}$$

(20) $$A_{2}=\pi r_{2}^{2}$$

(21) $$\frac{A_{2}}{A_{1}}=\frac{\pi r_{2}^{2}}{\pi r_{1}^{2}}$$

Then, for a purely geometric light propagation, the CPC brightness improvement is:

(22) $$\frac{90^{2}}{15^{2}}=36$$

However, when considering diffraction effect of the aperture:

(23) $$\theta =1.22\times \left(\frac{\lambda }{D}\right)$$

(24) θ=1.22×(0.525 µm/2.56 µm)

(25) $$\theta =30^{{}^{\circ}}$$

Then, the total angular distribution output is the sum of the diffraction angle and the CPC geometrical angular distribution:

(26) $$r_{1}^{\prime }=\theta \mathrm{diffraction}+\theta \mathrm{CPC}$$

(27) $$r_{1}^{\prime }=30^{{}^{\circ}}+15^{{}^{\circ}}=45^{{}^{\circ}}$$

In this case, the right improvement should be:

(28) $$\frac{r_{2}^{2}}{r_{1}^{\prime }{}^{2}}=\frac{90^{2}}{45^{2}}=4$$

Furthermore, since the CPC walls are made from a reflective material such as Sliver or Aluminum, additional absorption needs to be deducted to evaluate to total light efficiency. At a wavelength of 525 nm, Silver absorption is α = 1.7% [28] and Aluminum α = 8.3% [29].

#### 4.4.3. The Total LENA Pixel Efficiency

After evaluating the improvement generated by each part of the pixel, we can proceed to the total pixel light efficiency improvement. The total pixel efficiency is given by the equation:

(29) $$\begin{array}{l} \text{Cone LED LEE improvement}*\text{CPC brightness improvement}*\text{Alumium reflectance}\\ =10.43\times 4\times 0.917\\ =38.26 \end{array}$$

The Total improvement is 38 times. Meaning that, for the same electrical power consumption, the battery life duration is increased by 38! Furthermore, this improvement significantly decreases the operating temperature. Since the Cone LED has an LEE equal to 72%, only 28% of the light is absorbed in the LED structure and transformed in thermal radiation. In comparison for a flat LED 93.1% of the light is absorbed and transformed in thermal radiation. The light absorption in the LED is decreased by a factor of:

(30) $$\frac{\text{Flat LED light absorption}}{\text{Cone LED light absoption}}=\frac{93.1\%}{28\%}=3.325$$

It means that the thermal radiation is decreased by the same factor of 3.325.

Although by concentrating light in a useful manner to match an optical system numerical aperture, the battery consumption is lowered as explained earlier. This permits to further lower the thermal radiation by a factor of A_2_/A_1_, as seen before, by 4 in our study. The total pixel thermal radiation should be then lowered by a factor of 3.325 × 4 = 13.3. 

### 4.5. LightTools Parameters Summary

Since a lot of results were obtained, and presented above, it was important to summarize in one place all the parameters which were used for the 2D simulations. They are presented in Table 1.

## 5. Discussion

### 5.1. Simulation vs. Reality

Ray tracing is a geometric tool allowing the rapid tracing of the light path for narrow collimated beams called rays. Rays are randomly generated with a Monte Carlo algorithm and their behavior is fixed by Fresnel Descartes equations. Each “beamlet” called ray propagates into a predefined structure and encounters different material and surfaces. The main advantage of ray tracing is that ray paths are not pre-determined by the user. This permits discovering unexpected artifact like stray light and ghosts in imaging systems. Ray tracing software relies on perfect surfaces, ideal shapes, and material quality. Furthermore, physical optics phenomenon like diffraction effects and interferences require wave theory solving and are not completely or not at all simulated in Zemax and LightTools ray tracing software.

Several questions can be raised in light of the gap existing between simulations and reality. Real manufacturing implies tolerances and boundaries which are neither always well-known nor understood. Often, a deep investigation based on multiple tests and years of experience is necessary to know what parameters are more sensitive than others. As a result, any simulation made with theoretical assumptions is doomed to be not enough realistic and accurate. Then, it is hard to evaluate if the Cone and the CPC parabolic shapes can be manufactured as in our simulations, or if manufacture tolerances are reasonable. This should be eventually known and understood with manufacturing tests and experiences. Furthermore, the surface quality which is considered as ideal in simulations is obviously much more challenging in reality. Surfaces are naturally diffusing in a real word, where in theory we expect them to be specular. Controlling the surfaces roughness is another challenge to be taken into account in nano-metric and micro-metric scales. However, the main lack of consistence is regarding the edge’s diffractions of the pixel. As the pixel aperture is close to the wavelength, the light is scattered beyond the geometric specular distribution.

### 5.2. Rectangular vs. Circular Aperture Diffraction

When transmitted through a slit, light diffracts due to an edge diffraction effect. The pixel aperture shape has a direct effect on the diffraction pattern generated by light propagating through it. Indeed, as predicted by Bessel function, a circular aperture will generate a circular diffraction pattern. Instead, a rectangular or square aperture generates a central rectangular spot and a crossed pattern of the overall diffraction [30]. Most of the light is concentrated in the first central disk or rectangle. Indeed, 86% of the total diffracted energy pattern is inside the first dark ring. We now understand what far field light distribution could generate a rectangular LED aperture in comparison to our circular aperture shape. The central rectangular diffraction pattern has similar energy properties and could be a relevant shape to design a micro LED. The reason we chose to study a circular aperture is because its more conventional and symmetric shape properties. However, a rectangular shape could have its own advantages as a better fill factor of the pixel matrix. Indeed, filling a surface of little circles is much less efficient than with rectangles. This earlier shape could allow a much smaller spacing between two close pixels. The display resolution should then be increased. According to Joseph Louis Lagrange, the best method to pack circles in a Euclidean space is by arranging them in a hexagonal way. In this case, the packing intensity or fill factor equals ~0.9069 [31]. Still, a rectangular shape packing intensity equals 1 for obvious reasons. In ideal conditions, the pixel resolution gain is then ~9% for rectangular pixels micro LED.

### 5.3. Sub-Wavelength Aperture

Our study explores a pixel for which the aperture is larger than the wavelength. Further investigation could be done by evaluating a pixel with an aperture smaller than a wavelength. When light propagates in a sub-wavelength slit geometric consideration is not relevant anymore, as well as far field propagation. Indeed, in such a small scale, light interacts with matter atoms. Light propagation through a sub-wavelength slit is dictated by a Maxwell electromagnetic equation in matter. Maxwell equations take into account the slit thickness as well as the slit matter permittivity. In such a way, the plasmons influence on the light electric field is quantified and allows an accurate propagation model. The light transmission efficiency will directly depend on the physical interaction between free electrons in the matter and photons. Such phenomenon is known as the “plasmonic effect” [32]. Sub-wavelength LED aperture exploration may require physical additional simulations, and this is why complementary investigations will be set in the very near future. In this article, we focused on Ray Tracing and Efficiency.

### 5.4. Enhancement of the LENS Pixel Wall Reflection

**Figure 21:**
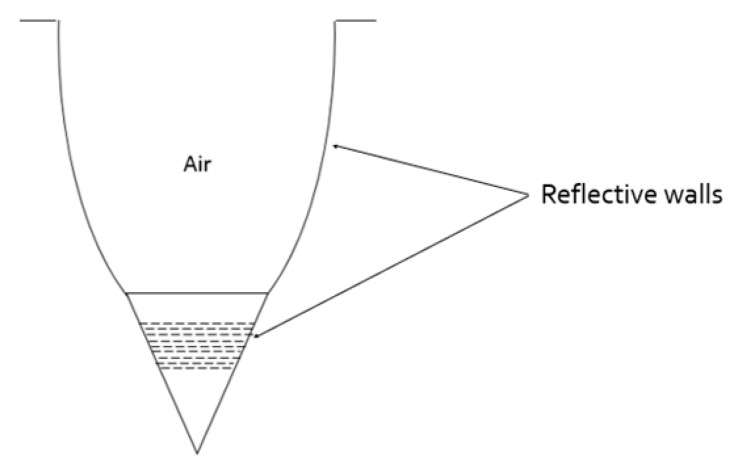
LENS pixel reflective walls.

As earlier described, the Pixel outer surface is reflective walls impeaching light to exit the pixel structure by the sides and redirecting rays to the top pixel output. In our simulation, we investigated mainly two different reflective materials, Aluminum and Silver. These two materials are commonly used in optics. However, these materials are not ideal and their reflectance is not perfect. As explained before, these materials absorb a small portion of the light decreasing the light efficiency in particular where multiple reflections are occurring. In order to decrease the light absorption from the reflective walls (Figure 21), two different solutions could be explored. The first solution should be to add a dielectric layer coating above the reflective material such as Aluminum. Such a configuration is known as “enhanced Aluminum” [33]. It improves the reflective property. The disadvantage would be that it requires a more complicated process by adding an additional step for the dielectric layer.

The other solution could be to abandon metallic material and instead to design a dielectric mirror coating. Metallic material advantage is their high performances over broadbands and large angular ranges. Designing a dielectric mirror coating made of multiple layers is a relevant solution for narrow angular range and/or narrow spectrum [34]. Since our spectrum is limited to the narrow LED spectrum one, such a dielectric coating mirror could be considered. Dielectric mirror coating has the potential to exceed metallic material reflectivity.

## 6. Conclusions

Summarizing the main achievements of this first research, several aspects of a new Light Emitting Nano-pixel Structure (LENS) were proposed, based on complementary studies and novelties, including concept, architecture, and analytical adapted models. A new concept of a self-emitting pixel was investigated. The pixel is a green light Nano LED with an optimized reflective conical shape. This shape allows Light Extraction Efficiency considerable improvement of 900% in comparison to a flat LED shape. The Nano LED aperture dimension is larger than a wavelength and is optimized to fit a light condenser element. A Compound Parabolic Concentrator, usually used to concentrate sun light into solar cells, is mounted on the cone LED to narrow the output light angular distribution. The mounted CPC succeeded in controlling the output Light angular distribution. The resulting intensity gain is 400%, aperture diffraction included. When combined together, it was demonstrated in our simulations that the cone Nano-LED and the Compound Parabolic Concentrator improve the power efficiency by around 3800% compared to a flat shape LED.

Regarding the prospect of further research into this topic, further additional steps can be considered to our study. Indeed, only the optical aspect was investigated and no anode and cathode were designed to allow a necessary electrons flux. Such an electronic design will permit considering a concrete development of the novel pixel. Thus, a manufacturing process flow could be created to ensure a feasible and optimized fabrication. Moreover, the self-emitting pixel design could be modified and adapted to manufacturing machines capabilities and realistic processes. The optical software used in this thesis is unable to predict the light behavior in sub-wavelength apertures. Smaller and smaller pixels are of obvious interest and physical software would contribute to a better understanding of sub-wavelength pixel efficiency and far field distribution. To finish, an array of pixels with a dedicated addressing scheme is the road leading to the ultimate new generation of ultra-high efficiency and ultra-high-resolution display. Complementary research, including next steps like physical behavior analysis, which is complementary to the ray tracing method, manufacturing, and testing will follow for sure, and we are already there.

## Figures and Tables

**Table 1:** LENS parameters using LightTools software.

Parameters	Parameters Definition	Values
**Device parameters:**		
D_em_	Emitting layer distance from top LED surface	42 nm
R_em_	Emitting layer Radius	287 nm
A_em_	Emitting layer Area	14.826 µm²
D_cbase_	Cone base diameter	45 nm
D_ctop_	Cone top surface diameter	664 nm
OHc	Cone overall height	442 nm
n_p-GaN_	P-GaN refractive index	2.45
n_n-GaN_	N-GaN refractive index	2.42
R_Alum_	Aluminum reflectance at 525 nm	91.703%
R_Silver_	Silver reflectance at 525 nm	98.341%
Ratio	Cone LED dimension ratio	2:3
D_cpcbase_	CPC base diameter	664 nm
D_cpctop_	CPC top surface diameter	2564 nm
OL_cpc_	Overall CPC length	6023 nm
InA	CPC Input Angle	90°
OutA	CPC Output Angle	15°
**LightTools setup parameters used:**		
LUM	Photometric Source flux	100 Lumen
λ	Wavelength LED emission	525 nm
σ_θ_	Emitting layer Angular distribution	Lambertian
**Measured parameters:**		
σ _ConeLED_	Output Cone LED angular distribution	Lambertian
σ _assembly_	Output Assembly angular distribution	±15°
LAl _ConeLED_	Output Cone LED light power with Aluminum reflective material	72.74 Lumen
LSi _ConeLED_	Output Cone LED light power with Silver reflective material	78.18 Lumen
L _assembly_	Output Cone and CPC assembly light power with Silver reflective material	76.88 Lumen
L _assembly_	Output Cone and CPC assembly light power with Al reflective material	66.70 Lumen

## References

[1] (2019). Global Market Shipment Share Held by LCD TV Manufacturers from 2008 to 2018. https://www.statista.com/statistics/267095/global-market-share-of-lcd-tv-manufacturers.

[2] Betancourt D., Del Rio C. (2006). Study of the Human Eye Working Principle: An impressive high angular resolution system with simple array detectors. Fourth IEEE Workshop on Sensor Array and Multichannel Processing.

[3] Lumus Transparent Displays. https://lumusvision.com/.

[4] A New Vision for Compting. https://www.microsoft.com/en-us/hololens.

[5] Guttag K. (2017). Near Eye Displays (NEDs): Gaps In Pixel Sizes. https://www.kguttag.com/2017/06/07/gaps-in-pixel-sizes.

[6] Theglobalscientist (2014). The Next Big Step in Computers: What Can Physics Offer Us?. https://theglobalscientist.com/2014/04/07/the-next-big-step-in-computers-what-can-physics-offer-us.

[7] Popular Science (2017). The Incredible Evolution of Supercomputers’ Powers, from 1946 to Today. https://www.popsci.com/supercomputers-then-and-now.

[8] Himax Color Sequential LCOS. http://www.himax.com.tw/products/microdisplay-products/color-sequential-lcos.

[9] Digi-Capita (2017). After Mixed Year, Mobile AR to Drive $108 Billion VR/AR Market by 2021. http://www.digi-capital.com.

[10] Carmigniani J., Furht B. (2011). Handbook of Augmented Reality.

[11] Carmigniani J., Furht B. (2011). Handbook of Augmented Reality.

[12] Eisenfeld T., Karsenty A. (2020). Super High Intensity Nano Emitting (SHINE) Pixel for High Resolution and High Brightness Displays. J. Nanophotonics.

[13] Liu J.-L., Zhang J.-L., Wang G.-X., Mo C.-L., Xu L.-Q., Ding J., Quan Z.J., Wang X.-L., Pan S., Zheng C.-D. (2015). Status of GaN-based green light-emitting diodes. Chinese Physics B..

[14] Eun L.H., Hong K.J. How Bright of Luminance Is Needed for Outdoor Commercial Display? 2016 IEEE 6th International Conference on Consumer Electronics—Berlin (ICCE-Berlin). https://ieeexplore.ieee.org/document/7684740.

[15] Herrnsdorf J., McKendry J.J., Zhang S., Xie E., Ferreira R., Massoubre D., Zuhdi A.M., Henderson R.K., Underwood I., Watson S. (2015). Active-MatrixGaN Micro Light-Emitting Diode Display with Unprecedented Brightness. IEEE Trans. Electron Devices.

[16] Carmigniani J., Furht B. (2011). Handbook of Augmented Reality.

[17] Robinson M.G., Chen J., Sharp G.D. (2005). Polarization Engineering for LCD Projection.

[18] Zhang Z., You Z., Chu D. (2014). Fundamentals of phase-only liquid crystal on silicon (LCOS) devices. Light Sci. Appl..

[19] Tanaka N. (2017). Sony’s OLED Micro-Display Realizes Both Small Pixel Pitch, High Image Quality. http://techon.nikkeibp.co.jp/atclen/news_en/15mk/062001307/?ST=msbe.

[20] Hornbeck L.J. (2002). Digital Light Processing and MEMS: An overview. Digest IEEE/Leos 1996 Summer Topical Meeting. Advanced Applications of Lasers in Materials and Processing.

[21] Hosseini P., Wright C.D., Bhaskaran H. (2014). An optoelectronic framework enabled by low-dimensional phase-change films. Nature.

[22] Ra Y.H., Wang R., Woo S.Y., Djavid M., Sadaf S. Md., Lee J., Botton G.A., Mi Z. (2016). Full-Color Single Nanowire Pixels for Projection Displays. Nano Letters.

[23] Basak S., Mohiddon M.A., Baumgarten M., Müllen K., Chandrasekar R. (2015). Hierarchical multicolor nano-pixel matrices formed by coordinating luminescent metal ions to a conjugated poly(4′-octyl-2′,6′-bispyrazoyl pyridine) film via contact printing. Nature Sci. Rep..

[24] Wang S.W., Hong K.B., Tsai Y.L., Teng C.H., Tzou A.J., Chu Y.C., Lee P.T., Ku P.C., Lin C.C., Kuo H.C. (2017). Wavelength tunable InGaN/GaN nano-ring LEDs via nano-sphere lithography. Nature Sci. Rep..

[25] Djuriie A.B., Chan Y., Li B.H. (2001). Calculations of the refractive index of AlGaN/GaN quantum well. Proc. SPIE.

[26] Born M., Wolf E. (1970). Principles of Optics: Electromagnetic Theory of Propagation, Interference and Diffraction of Light.

[27] Sawant S.Y., More S., Rvindranath G., Thipase S.S. (2018). Mathematical modeling and analysis of compound parabolic concentrator using soltrace. Int. J. Mech. Eng. Technol. (IJMET).

[28] Johnson P.B., Christy R.W. (1972). Optical Constants of the Noble Metals. Phys. Rev. B.

[29] Rakic A.D. (1995). Algorithm for the determination of intrinsic optical constants of metal films: Application to aluminum. Appl. Optics.

[30] Born M., Wolf E. (1970). Principles of Optics: Electromagnetic Theory of Propagation, Interference and Diffraction of Light.

[31] Chang H.C., Wang L.C. (2010). A Simple Proof of Thue’s Theorem on Circle Packing.

[32] Schouten H.F. (2005). Light Transmission through Sub-Wavelength Apertures. Ph.D. Thesis.

[33] Baumeister P. (2004). Optical Coating Technology.

[34] Harry G., Bodiya T.P., DeSalvo R. (2012). Optical Coatings and Thermal Noise in Precision Measurement.

